# Let's Get Back to Normal? COVID-19 and the Logic of Cure

**DOI:** 10.3389/fsoc.2022.782582

**Published:** 2022-04-12

**Authors:** Maria Berghs

**Affiliations:** Allied Health Sciences, De Montfort University, Leicester, United Kingdom

**Keywords:** cure, sociology, COVID-19, anthropology, pandemic

## Abstract

The COVID-19 pandemic has inversed certainties of absolutes of cure in everyday life but paradoxically this has occurred during a time when novel scientific advancements seem to herald a new frontier of cures for rare diseases, chronic conditions, disabilities and viruses that were previously incurable. In this paper, I illustrate the development of a logic of cure by first of all noting a lacuna in the medical sociological and anthropological literature, where although a lot of empirical research and theoretical work to understand cure has been undertaken, there has been no sociology or anthropology of cure. Using three case studies, I examine what they reveal about the logic of cure. Firstly, I argue that there is a development of a bioethics of cure in reactions of disability community and disabled people to care as cure during the COVID-19 pandemic. The second case-study focuses on understanding limitations of vaccines and how people react against such indeterminancies of loss of absolutes of cure. Lastly, the final case study describes how while there are cures, for example, for rare genetic conditions, they are often initially curated with long-term cost-benefit analysis for the Global North. In conclusion, it is found that many of the developments within sociology and anthropology are missing from a logic of cure and that a new theory of cure has to develop.

## Introduction

The COVID-19 pandemic has brought to the fore the importance of critically examining our responses to accelerated scientific developments of “cures”. From the differing socio-cultural reactions in the Global North encompassing broad calls to get vaccinated and back to “normal” life to the hesitancy of risk and historical suspicion of scientific experimentation in some communities. As well as lack of choice of “cures” in inequalities in who gains first access to diagnostics, medicines, therapies and vaccines in the Global North and South, the pandemic has revealed not only the complexities of a curative imperative but also how the general public has coped with a loss of certainty of what “cure” now means.

In popular culture, what it meant to be incurable or have no possibility of recovery or cure, used to be reserved for serious physical or mental illnesses, chronic conditions, rare diseases, disabilities or viruses. The realm of everyday health and “normal” was not part of such medical pathology and environmental risks, thus “cure,” recovery, healing or convalescence from an illness or disease to “health” (Francis, 2022), did not need to be unduly examined. The very idea that there could be different forms of “cure” or of ways in which your body and mind recovered was never part of that analysis. Nor did cures encompass disability, disease or mental illness. Or even it's opposite, in temporal understandings of life where cure was never a given (Kafer, 2013; Clare, 2017). The emphasis has always been on the “curing” or how people recovered and were cared for, without description of the diversity of experiences of cure itself. Cure was always seen in terms of an end-point, rather than along a continuum of constant illness and possible or impossible recovery cycles, each with differing forms of cure during the life-course.

Despite warnings from the scientific community of future possibilities of anti-microbial resistance and epidemics of Zika, MERS-Cov and Ebola destabilizing our understandings of cure, illness and disability; that there would always be cures and recovery to health in the Global North seemed to be a popular given. While several successful vaccines have been found for COVID-19 and vaccination campaigns begun in many countries, this has initially been mainly in the Global North with localized priorities and vaccine nationalism often triumphing over global rights to health. Reactions to vaccine developments have also been dampened by noting how population engagement is critical, immunity might not be experienced similarly and that new variants of the SARS-CoV-2 virus may develop, which will again need fast vaccine development or vaccine boosters.

The COVID-19 pandemic has thus inversed certainties of absolutes of cure in everyday life but paradoxically this has occurred during a time when novel scientific advancements seem to herald a new frontier of cures for rare diseases, chronic conditions, disabilities and viruses that were previously incurable. For instance, innovative developments in vaccines can now target the structure of immunogen designs to ensure immunity against influenza, gene-based vaccine platforms like mRNA can target respiratory syncytial virus, and using recombinant proteins can ensure control of latent tuberculosis infection (Mascola and Fauci, 2020). Similarly, the development of the Moderna mRNA vaccine has had a ripple effect in causing research excitement that a successful vaccine for HIV could be developed using the same insights (Esteban et al., 2021). Some of these current developments build on scientific and technological advances, for example in genomics and genetics, that mean a better understanding of diseases, infections and their prevention. Due to the increase of high-dimensional biology, omics testing (genomics, proteomics and metabolomics), screening services, use of big data analytics, increase of biomonitoring and the move toward personalized medicine based on genes (genomics), mRNA (transcriptomics), proteins (proteomics) and metabolites (metabolomics), there are advances in early identification, prevention as well as innovation of technologies of cure. For example, in pharmacogenomics, direct to consumer genetic testing or the understanding of how inheritance can predict biological responsiveness to particular medicines (Horgan and Kenny, 2011; Alyass et al., 2015; Li et al., 2016; Dennis et al., 2017).

In the above, a “logic of cure” that places as central an imperative to cure, which has become normalized and commodified is evident. Just as Mol (2008) contrasted a patient centered “logic of care” with one of consumer “choice” in medicine, I argue that these scientific and technological advances have divested both care and cure to the needs of the marketplace. While the material of the human body seems to be at stake, cures bring into focus how the biological, socio-cultural and environmental become entangled because of their economic and political impacts. Hence, Niewöhner and Lock (2018) argued that with epigenetic advances, there had to be recognition of how the relationship between nurture and nature had changed in situating local biologies. The neoliberal promises of late modernity have been incorporated into such a curative imperative and patients, health care professionals, industry, academic as well as philanthropic investors now act together with politicians and national interests to ensure economic, political and socio-cultural momentum around developments of cures, such as during the COVID-19 pandemic. This reveals stratified inequalities in not only what will be cured but also who, when and where curative processes will take place and why.

In this paper, I illustrate the development of a logic of cure by first of all noting a lacuna in the medical sociological and anthropological literature, where although a lot of empirical research and theoretical work to understand cure has been undertaken, there has been no explicit sociology or even anthropology of cure (Berghs, 2021). Using three case studies, I examine what they reveal about the logic of cure. Firstly, I argue that there is a development of a bioethics of cure in reactions of disability community and disabled people to care as cure during the COVID-19 pandemic. The second case-study focuses on limitations of COVID-19 vaccines and how people react against such indeterminacies of cure. Lastly, the final case study describes how while there are genomic cures, for example, for rare genetic conditions, they are often curated with cost-benefit analysis. In conclusion, it is discussed what the three case studies reveal about the development of a sociology and anthropology of cure.

## Why No Sociology of Cure?

If you examine the plethora of sociological and corresponding philosophical and ethical literature on care, there has been work on “cure” but it seems odd that cure has not been given the same explicit attention as care? For instance, there is no philosophy of cure but a philosophy of care is a well-grounded discipline. Despite the proliferation of the term cure and its link to care in clinical terms, not much has been explicitly written in sociological research theoretically investigating cure. What an accelerated curative imperative now entails? How that feels as a patient or for a family if you have had a life-long or life-threatening illness and been “functionally” cured? What technologies and commercial investments become implicated, in what biological materials, and if and how the future changes if your “curative hopes” have been realized or ruined? Generally, there is a lacuna about “cure” but that is correlated to how we understand cure as becoming ever more specialized in particular therapies, medicines and interventions. Cure is also scientifically developed as innovative “hype” to be critically sociologically appraised, in keeping with new understandings of self, body and environment developed from the 1980s onwards.

## Understanding Ourselves?

Sociological lay understandings and experiences of health and illness from the 1980s onwards have typically focused on narratives of loss when faced by illness, disease and disability. For instance, in “biographical disruption” (Bury, 1982), “loss of self” (Charmaz, 1983) to “narrative reconstruction” (Williams, 1984), and the ways in which lay understandings of the “self” are confronted with biomedical explanations of illness, disease and disability when there is no cure (Lawton, 2003). Furthermore, conceptions of the body, selves and embodiment underwent further rapid changes from medicalization, (bio) medicalization to genetization with the start of the 1999 Human Genome Project (Wolputte, 2004). However, Lippman's (1992) “genetic imaginary” thesis warned how genetic explanations would become dominant to our future understandings of human health and behavior in society, noting ethical and eugenic concerns. The 1990s also saw the rise of surveillance medicine (Armstrong, 1995), the idea of genes as embodied risks (Hallowell, 1999) and new forms of somatic citizenship that were based on genetic understandings of the self (Novas and Rose, 2000).

These ideas built on Rabinow's (1996) insights that argued that new forms of social relations based on biology would form in terms of “biosociality” or biosocial identities of groups of patients. Near the end of the Human Genome project, there was a lot of interest in its' implications, for instance, in prospects of gene therapy for cures of rare diseases (Stockdale, 1999). However, there were criticisms too as Williams (2000) explained how such genetic explanations of self and chronicity of the body were problematized and pathologised. We also noted a reaction in sociological literature against “speculative” scientific cures in the popular press, and warnings about “hype” and economy of raising false hopes (Brown, 2003). The sociology of disability also elucidated the difference between biological basis of impairment and experience of disablement by society, noting the social construction of “disability” (Timmermans and Haas, 2008) or how biological forms of impairments could lead to social disablement.

The best version of the self was biologically “healthy” and Lupton (1997) argued that an “imperative of health” was established - of not needing curative intervention - which became embedded in our social and cultural norms. Hacking (2006, p. 81) described how this had progressed toward a “genetic imperative – the drive to find biological but above all genetic underpinnings for all things human, in sickness or in health”. Within the literature and society in general, there was a continued engagement with geneticization in the 2000s which heralded more uncertainty linked to the increase of genetic explanations in medical, ethical and social discourses (Hedgecoe, 2003). Lay perspectives became gendered as women had to become “moral pioneers”, through engagement with increasingly invasive technologies of screening in antenatal care (Rapp, 2004), genetic testing normalized and reproduction stratified in terms of inequalities (Lock and Nguyen, 2018). There were also calls for a sociology of health, illness and disease, to examine how biology and disease were intertwined (Kerr, 2004; Timmermans and Haas, 2008). Despite warnings about the dominance of the genetic imaginary, Weiner et al. (2017) argued that genetic explanations did not impact day to day life as much as would have been expected but also that those genetic imaginaries were now changing into molecular or pharmaceutical explanations.

Genetic understandings of embodiment also gained more complexity with epigenetic explanations, as there was not always a clearly defined path identified between disease and genetic expression of an illness (Shostak et al., 2008). Shildrick (2010) argued that neither biomedical nor socio-cultural explanations of a bounded body seemed to hold sway. Yet, the clinical diagnosis of disease and biological understandings remained important to lay people, and patients demanded to talk about their experiences of diagnosis and we saw the beginnings of a sociology of diagnosis (Nettleton, 2006). As genes became expressions of neoliberal risks and parts of the body commodified (Sharp, 2000), research began to examine ever smaller biological units of bodily consumption and literature on the bioeconomy developed (Birch and Tyfield, 2013). Bioeconomies were associated with the rise of genomics and epigenetic advances which also entailed a paradigm shift in biomedicine away from genes to molecular biology and environmental determinants (Stoneking, 2016). According to Arribas-Ayllon (2016) the way in which to think of how this worked was not a constructionist approach but rather that of understanding “assemblages” of “genes, people and environments” (Hacking, 1999; DeLanda, 2019).

Ontologies and epistemologies have typically focused on the rise of biological realism and materialisms, in terms of genetic diagnosis and linked technologies such as, for example, the CRSPR-Cas9 gene editing technique. Thus, Lock et al. (2015) warned against the collapse of the nature and culture divide in epigenetic explanations and rise of neo-reductionism or reduction to biology. However, it was argued that while health is becoming increasingly biomedicalized, rather than a vision of biological essentialism, research revealed shifting conceptions of identity but also wider biological understandings of the body as assemblages of (micro) chimeras, bacteria, viruses and even food (Waldby and Mitchell, 2006; Martin, 2007; Shildrick, 2010; Landecker, 2011; Fritsch, 2016; Gibbon et al., 2018; Hinterberger, 2018). These unsettled the epistemological and ontological lines between human and non-human and how we understood who we were and in turn, what rights and responsibilities we accord ourselves and the environment. Moreover, it was becoming increasingly difficult to think humans without non-human symbionts (See Morton, 2017), with arguments advocating understanding epigenetics, impact of intergenerational historical trauma on biology and future outcomes, in what Ingold and Palsson (2013) term “biosocial becomings” - to illustrate how the biological and the social, or nature and culture, are tied together. Yet, such arguments were often not reflected in the way in which personalized medicine or health surveillance was understood in practice.

Health surveillance became more encompassing, for instance, through (bio) digitalization and quantification of health in almost every aspect of daily life, as well as social understandings with respect to digital technologies (Lupton, 2012). Classifications of illnesses, disorders, diseases and disabilities require differing forms of bio-technological diagnosis and clinical identification or biocertification (Samuels, 2014; Fritsch, 2016) before a “cure” could be found. This has implications for identity and social relations, in that diagnosis or biocertification seemingly “fix” a biomedical identity or label it. In such a way, the cause and also the cure must be found in biological explanations, as we see, for example, in connections between genetic explanations for some psychiatric conditions (Arribas-Ayllon et al., 2019). Personalized medicine has meant a rethinking of disease taxonomies for those that are genomics based, but which has been difficult to implement in clinical practice due to uncertainties of genomic variation (Green et al., 2019; Milne, 2020). Despite this, new forms of biosociality are thus developing around more specific forms of biological data, toward biosocial potentiality, like the routine collection of biomarkers in social surveys as “precursors” of disability (Davillas and Pudney, 2020) or “exposomic” biological monitoring for environmental exposures (Dennis et al., 2017).

Thus, Rose (2009) argues that we are witnessing a new form of active “global genomic citizenship” which accepts neoliberal forms of individual consumer responsibility for understanding risks of disease in our genomic data. Aligned is the idea that pathologies in genomic data is the new norm (Rose, 2009) and we are all genetic carriers of various risks. We now have to work at being and acting healthy which can involve: consuming certain foods; vitamins or superfood powder; exercise, medication; undertake various types of screening; engage in stress free social relations; as well as the routine collection of differing forms of genealogical and lifestyle data. An associated issue is this norm of hyper-engaged health, assumes that there is a hierarchy of health and able body, which we are and can be individually responsible for to prevent health risks. In such a way, the imperative of health, or norm of caring for ourselves by undertaking healthy “choices”, is also increasingly becoming an imperative of curing oneself and a new form of individual and collective public health and “body work” (Kerr, 2013). This kind of “body work” has an enduring correlation to health – to keep “fit” and “fitness” as industry (Maguire, 2007) as well as reproduction and fertility. For example, claims become correlated to perceived “fitness” to be able to mother and father a child (Hanna et al., 2018), which is also increasingly about genetic future fitness and social potentialities (Arribas-Ayllon et al., 2019).

**Figure 1:**
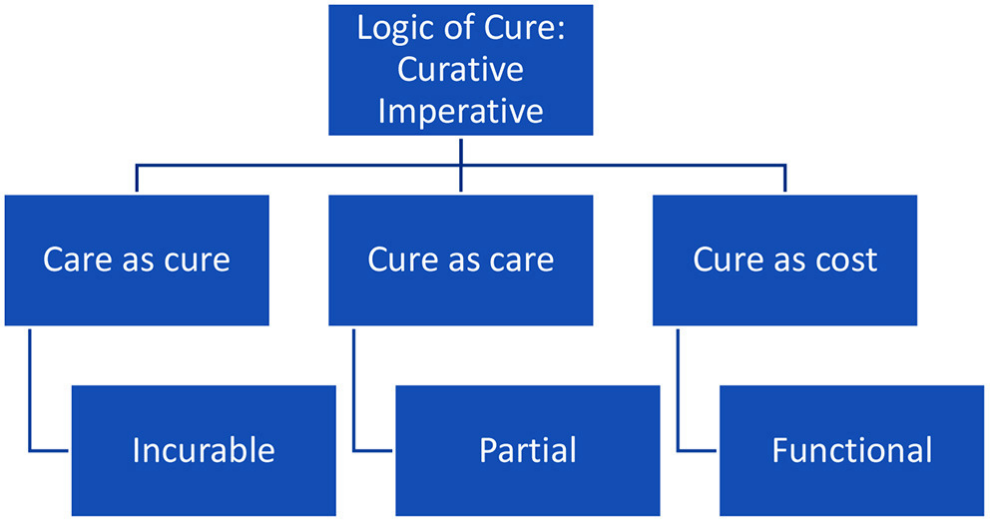
The logic of cure.

In the brief overview above, there is a tension between the indeterminacy and ever complexity of understandings of human selves and how curative potentials become implicated. This partly explains why we do not focus on “cure” as a general concept, as research on diagnosis, personalized medicine and what happens to identity or in the clinic while cure takes place are prioritized. However, this research occurred before the COVID-19 pandemic and in what follows, three examples are given to illustrate the logic of cure as it functions today and what that reveals. We look at care as cure, cure as care and cure as cost (see Figure 1).

## Case-Study 1: Disability and the Pandemic: When Care Means Cure

What does a neoliberal responsibility to cure oneself look like and is it always possible in practice? Discourses linked to fitness, cure and medicine, as well as contestations to genomic identity are not viewed neutrally by disabled people, those with chronic illnesses nor their organizations, who are often critical of technological advances to “cure” all (Hughes, 2009) but can also biosocially ascribe to medical identities or impairments due to the hopes of cure (Ottosdottir and Evans, 2016; Berghs, 2020). Within disability community, alongside such complexities, there have also been longstanding concerns about soft eugenics in screening technologies to prevent disability, the ideology of “ableism” or having to conform to ableist physical norms of health (Campbell, 2009), and violence of medical cure and “curative imaginary” in terms of threat to the integrity of the body, “crip time” and disability identity (Kafer, 2013; Kim, 2016; Clare, 2017).

Disabled people and disability activists often argue against (bio) medical or genetic understandings of disability, or biological essentialism and reductionism, instead pointing to social barriers to inclusion and equality, in keeping with social becomings or a social model of disability. The social model of disability makes a distinction between biology of impairment and disabling experience of social oppression through negative social attitudes and/or environmental barriers in society (Oliver, 1983, 2013). It also draws attention to the inequalities across the life-course connected to both gaining impairment and experiencing disablement (Oliver, 1983, 2013). An important aspect of such inequalities has always been a lack of access to health and social care. The social model of disability is also foundational to human rights approaches to disability which view access to health, and thus healthcare, as a human rights issue (Shakespeare, 2015).

The COVID-19 pandemic illustrated many of the complexities of the relationship between disability and what a curative imperative now implies, in how healthcare access initially prioritized able-bodied health as norm in state policy (Goggin and Ellis, 2020). As COVID-19 began affecting disabled people in care homes and locked institutions with discussions of medical rationing of vital equipment, such as oxygen and access to medicines in emergency situations, the focus turned to questioning medical ethics and (bio) ethical responses with utilitarianism seemingly trumping over not only the sanctity of life principles (Sabatello et al., 2020), relational care but also rights based approaches to healthcare. The lack of access of basic health care was touted as disablist (Scully, 2020) and the triaging or giving of priority to able-bodied and healthy people to receive therapies that could cure, ableism (Campbell, 2009). Yet, at the heart of such policies was an understanding of “recovery” or who would be curable which is different from ableism, in that it is not just able body that is prioritized but temporal ability to stay well. Initially, policy responses benefited the able-bodied majority and while purporting to protect the “vulnerable”, policies caused disabled people to begin to experience risks to COVID-19 (Glover et al., 2020). Policy decisions also lacked inclusion of disabled people, their organizations or basic accessibility, such as government announcements that were inaccessible, for example, by holding televised government broadcasts with no sign-language interpreters in the UK.

Due to inadequate Personal and Protective Equipment (PPE) in hospitals and residential facilities, lack of accessible information and the discharging of people from hospitals into care homes and residential facilities, particular disabled groups were more at risk for COVID-19 and dying early on in the pandemic. These included older people, those with co-morbidities and people with learning difficulties and autism. Those were disabled groups that were particularly discriminated against within health and social care systems and had been neglected pre-pandemic (Inclusion Europe, 2020). Intersectionality also began to affect who was most at risk from COVID-19 with inequalities revealed in gender, ethnicity, impairments, age and socio-economic status in who was impacted directly and indirectly. Despite COVID-19 being linked to inequalities and deprivation, (bio) medical model discourses around “underlying conditions”, “genetic dispositions” or biological “racial” risks began to take precedence over explanations of structural disadvantage or syndemics (Boulware, 2020; Gravlee, 2020).

Vulnerability to impairments and deaths that were created in the COVID-19 pandemic illustrated dangers of narrow understandings of cure and (bio)ethical responses that only focused on care or rights. Arguments about biological essentialism and dangers of bodily reductionism, despite epigenetic complexity of how we understand our “selves” sociologically and anthropologically, proved to be persuasive in practice. Likewise, the reductive genetic imaginary of people's biological potentialities or dispositions for cure instead of ethical inclusion and rights of equity of treatment (Armitage and Nellums, 2020), were foundational not only in popular discourses but in preventing access to medical treatment. Noteworthy, is the rise of an encompassing biosocial model of disability (Berghs, 2020) that now affects everyone because it is predicated on epigenetic risk and biological impairment. In the next example, we look at the curative hopes of the general population and how inequalities become stratified in vaccine nationalism and priorities of vaccination during the COVID-19 pandemic.

## Case-Study 2: Learning to Live With Indeterminacies of Cure: COVID-19 Vaccines and Cure as Care

Patients increasingly advocate for diagnosis, better care and also *rights* to cures (Novas, 2006) and care, in what Jae (2018) terms “anticipatory politics”. Curing is bound to the political economy of hope (Novas, 2006) and hopelessness (Coyle and Atkinson, 2018), as well as transnational promises of the genomic technologies of cure and evidence-based health activism and experimentation (Weatherall, 1990; Bharadwaj and Glasner, 2008; Rabeharisoa et al., 2014). Curing now also has an imperative of preservation or repairing of health as site of norm of life which is why “getting back to normal” featured in popular discourses around the race for vaccines for the SARS-CoV-2 virus that causes COVID-19. Despite sociological warnings of “hype” or anticipation of new treatments and unfulfilled promises in expectations of normalcy (Brown, 2003), hopes of new forms of treatment that *will cure* or prevent illness will dominate patient, public and technological discourses of innovation. Actions, emotions as well as technological developments for cures are temporally directed toward “promissory futures” (Brown, 2003), “curative imaginaries” (Kafer, 2013) or “imagined” futures free of viruses (Van Loon, 2013), which a global community is politically, social, culturally and financially invested in. However, such speculative futures become problematic if the general publics' conceptions of cure do not tie in with indeterminacies on offer.

Mass vaccination programmes are a given in many countries in the Global North with Pfizer/BioNtech, AstraZeneca/Oxford, Janssen/Ad26.COV 2.S, Moderna mRNA and Sinovac-CoronaVac among the vaccines being given to populations. However, while most of these vaccines could not offer absolute cures, they seemed to be presented in terms of reduction of total risks and representing individual and national forms of viral sovereignty linked to state political and economic interests (Van Loon, 2013; Yu, 2013). Within many of the COVID-19 pandemic vaccination campaigns, it is clear that cure is still understood as “absolute” for the general population. This meant offers of multiple vaccine doses and promises of getting back to a normal that did not initially materialize, were treated with suspicion, hesitancy and disbelief. Uptake of vaccines were also dependent on how cure was linked to experiential understandings of other illnesses and viruses and their risks along an affective and temporal pandemic continuum that ebbed and flowed (Caserotti et al., 2021). The risks and fears of safety of vaccines expressed by populations also often implicated many of the changing conceptions of the self and embodiment found in sociological and anthropological research, but this was generally ignored or just seen as misinformation about vaccines (Lockyer et al., 2021). For instance, some refusals of vaccines encompassed ideas implicit in healthism, the body as being fit, having immunity and being able to “cure” itself which have been very popular in the health, wellness and fitness industries used to sell products to consumers.

While citizenship and risks were connected to neoliberal individual responsibilities for consumer choice, there were no real informed choices over vaccine uptake, as people could not weigh individual experiential risks, for instance of side-effects, impact on co-morbidities or other conditions, nor what level of immunity a vaccine would give, by choosing which vaccine to have. Instead appeals were made to engagement of cure as care and ethical responsibilities to protect fellow citizens. Those kinds of sentiments while important, might be at odds with neoliberalism (or even libertarianism) focused on individual and consumer choice. Similarly, such appeals may not work for populations who have experienced historic and present inequalities of care, lack trust in their governments and/or suffered abuses from the medical establishment. While wanting to care for the self and others, some communities may also feel as if they are taking part in an experiment, feel hesitant about lack of informed choice or want more evidence of viral effects before taking up the vaccine offer (Calnan and Douglass, 2020; Lockyer et al., 2021). This is normal when none of the differing vaccines could promise a hundred percent immunity to the SARS-CoV-2, with reports in the press and on social media often weighing vaccines up against each other, leading to popular reports of vaccine envy, dissatisfaction and regret over lack of choices. It is crucial to note that this is in the context of vaccines presented as “cures” with a history of economic and social sacrifices as well as deaths from COVID-19. There was also discussion of long COVID in the popular press, as well as possible future of COVID-19 variants. In such a context, it seems entirely reasonable that people would want and need to make an informed choice about choice of vaccines and this could be emotional. This also changes the way in which public health measures are viewed because if we have vaccines presented as “cures” then that must mean that the collective body and state are “back to normal” and health? Even if vaccines are understood, the idea of “herd immunity” might also mean that certain public health measures are no longer acceptable, regardless of what the evidence or politicians might say.

Undoubtedly, while many people were also grateful and happy to take up vaccine offers as responsible citizens, there was ambivalence toward not only indeterminancies of cure presented but also with regards to local, national and international inequalities and curative ethics (Lockyer et al., 2021). All over the world people had died or had gained impairments, and in the Global North, consistent with structural disadvantage and inequalities, it was particularly ethnic minority communities that had been badly affected by COVID-19 (Lockyer et al., 2021), which led to the pandemic being understood as racialised. It was also mainly richer countries in the Global North, like the UK and United States (US), that engaged in vaccine nationalism rather than vaccine equity (Katz et al., 2021), often prioritizing national interests over global wellbeing. In countries like Brazil and India, there have also been accusations of curative fascism in lack of national planning to ensure that the population could get access to vaccines and have rights to cure. Within a global curative imaginary, access to vaccines represented survival not the “getting back to normal” of the Global North. While there are programmes such as COVID-19 Vaccines Global Access (COVAX) to ensure equitable global vaccine distribution, directed by GAVI, the Vaccine Alliance, the Coalition for Epidemic Preparedness Innovations, and the World Health Organisation (WHO), such international partnerships seemed to have to sucede urgency to national priorities which also protected pharmaceutical investments and distributions in the Global North (Tatar et al., 2021).

This case-study reveals how national interests and short term visions of quick fixes and partial curative hypes have a stronger hold than human rights to health, global ethics of care and international treaties or agreements. Such short-term visions when it comes to cure and care are also dangerous, not only in the affective experience of ambivalence over having vaccines, public health measures not seen as needed, as well as leading to development of new variants of the COVID-19 virus that might be more virulent and possibly evade the vaccines that have been developed. To date there have been several variants but none that have evaded vaccine development. However, for instance, both Delta and Omicron have been very transmissible and lead to hospitalizations of unvaccinated people and some vulnerable members of the population. This has brought ethical questions to the fore in how far people's freedoms not to choose to be vaccinated or cured can be understood, especially if it could lead to their hospitalization, them gaining impairments or even causing their deaths or those of more vulnerable members of a community (Del Rio et al., 2022). As such, some workplaces and countries have insisted on vaccines as mandatory, especially as certain public health measures become unacceptable and risks of transmission can no longer be contained but also may not need to be feared with vaccination.

As such, the vaccine response, so far, illustrates the limitations of curative hopes and how important it is to be transparent and explain what kind of cure is on offer, for example, that it will be partial, need top-ups and is not absolute. It also takes seriously that populations may have sophisticated ideas about how the biological and human environment become implicated in cures and understandings of viruses. This entails that discourses around COVID-19 should now include a new normal where learning to live with increased risks and having to top up vaccines each year is part of curative trajectories. This would give strength to understanding why cure as responsible act of citizenship and care for each other then becomes important. However, what is missing is an understanding of how unequal participation in cures ties into ethical ambivalences around racialised inequalities.

## Case-Study 3: Rare Diseases and Weighing the Costs of Therapies or Cure: Choosing Cures - Business as Normal?

It seems as if cures initially mainly benefit certain populations in the Global North but is this always the case? The transnational nature of seeking cures in both proven and unproven treatments as well-curative experiments is only expected to grow with the “genetic imperative” (Hacking, 2006) and will lead to difficult decisions for disease and patient organizations and community groups, in terms of understanding and delineating between evidence of treatments or trials that are legitimate and regulated; and those that do not work or are speculative (Petersen et al., 2015; Song, 2017). Jae (2018) argues that “structural conditions also stratify expectations for the future, including the affective appeal of medical innovations” concerning what treatments or trials for cure are available and what rate of success they have. Kato (2018) too explains that while genomics becomes tied to cure, nuances have to be made in how patients understand and want cures, with regards to present histories of discrimination, impact of racism, lack of care and scientific understanding about genomic medicine. In many ways, a new language of choices of cures and therapies that takes into account such concerns is needed and developing.

If we take “rare” diseases, such as, for example the inherited genetic blood disorders sickle cell and thalassaemia that affect millions of people worldwide, recent innovations in terms of therapies and curative genomic realities in gene editing (Frangoul et al., 2021) with “functional cures”, convey numerous difficulties in a global transnational context when we think of hopes of cure. While these conditions affect millions worldwide, in the UK they mainly affect ethnic minority populations, thus national classifications of how “rare” the respective conditions are and their applications, or scalability of those innovative cures for other common conditions, are key (Milne, 2020). Those considerations around therapies and curative realities also occur against the background of history of curative neglect and racialised pandemic where people with such rare diseases are identified as “clinically extremely vulnerable” (CEV) and have been encouraged to get vaccinated because they are at higher risks of morbidity and mortality due to their conditions and structural inequalities affecting them as minorities. What does cure mean against such a background and how are the costs and risks of cure weighed up and by whom? What does that mean for a patient's individual choice of cures and therapies?

In the UK, screening services in antenatal care, genetic testing, treatments such as hydroxyurea for sickle cell, blood transfusions for both sickle cell and thalassaemia, specialized clinical care and public advocacy have entailed that the conditions can now be viewed as chronic and long-term, whilst in many other places in the world, without access to adequate healthcare, they can be acute and disabling. In the Global North, innovative genomic developments have entailed a shift of research attention so it is focused on improvements in access to therapies, as well as curative possibilities for those with differing expressions of these genetic disorders (Jae, 2018). Stem cell transplants for children with the most serious clinical manifestations of the conditions, often from a genetically matched relation such as donor sibling, is an option, in terms of cure, but was not without serious risks, complications and mistrust. This has often raised difficult ethical, social and emotional issues about how to make and share these decisions within families (Sullivan et al., 2018) and what the rights are of older patients facing more serious manifestations of the conditions to such risky or newer experimental cures. A politics of social justice, “race” and health equity in terms of access to cures in resource poor settings in the UK, US and countries in the Global South where most people are affected; like in Nigeria, India and Brazil, that do not have access to the basics of good care, has also emerged (Bliss, 2012; Benjamin, 2013; Berghs et al., 2020).

**Figure 2:**
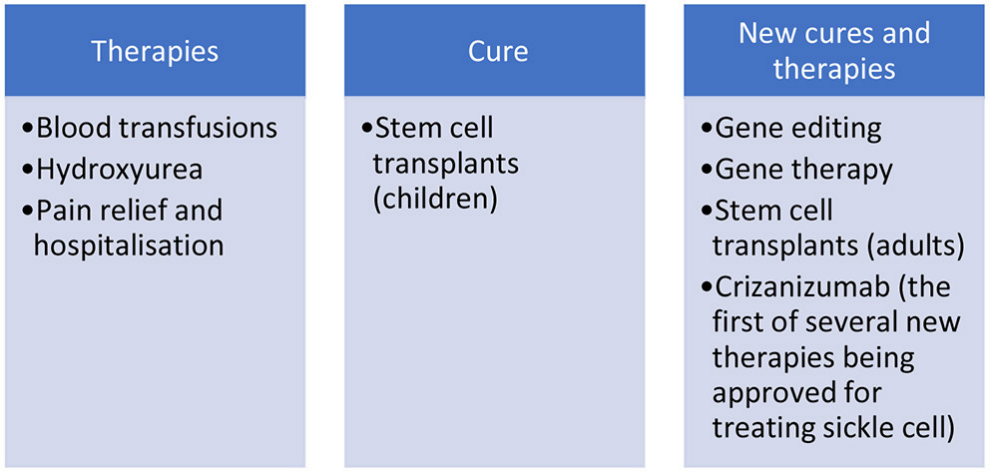
Simplified version of therapies and curative options for sickle cell condition.

The experimental possibilities and understandings of cure have changed with the advent of genomics and personalized medicine (Benjamin, 2013), making real immunotherapeutic possibilities such as gene editing out the mutation, stem cell transplantations in adults without need for chemotherapy or related donors, and gene therapy to change the affected genes (Urnov, 2021). Many of these experimental possibilities of cure are being tested in countries in the Global North in randomized controlled trials (RCTs) and have reversed the trajectory of ethical, relational and social dilemmas, for example, with respect to questions about if children should also have access to these experimental treatments and how decision-making should then occur (de Montalembert et al., 2017) and finances be found (Urnov, 2021). Alongside such experimental treatments for long neglected conditions, are hopes invested in new forms of therapies and medicines that have been developing that might mean less curative risks and increasingly manageable long-term conditions. For instance, Crizanlizumab has recently been approved as a new therapeutic treatment in the UK for patients with sickle cell. This is the first one in over 20 years and further therapies are in development and undergoing trials. This raises the critical question of who “curates” or who gives access to cures and why? And can patients have choices between cures and therapies? (see Figure 2).

For instance, in the UK recently, the National Institute for Health and Care Excellence (NICE) has not recommended the use of Bluebird Bios' Zynteglo (betibeglogene autotemcel) gene therapy for the treatment of patients with transfusion dependent beta-thalassaemia and is undergoing its appraisal. The national, and in particular the international patient support group, Thalassaemia International Federation (TIF, 2021), quickly reacted against the decision, noting how this was based on an understanding of thalassaemia as a manageable chronic condition with a good quality of life and life expectancy, which ignored the day to day difficulties and accumulative impact of the condition on impairment. Additionally, they argued that NICE weighed the short and especially long-term risks to the relatively small cohort of patients that would benefit, as well as cost-effectiveness of such a treatment to the NHS but TIF (2021) noted that for some patients those long-term risks of an uncertain therapy would be justified and that NHS estimations of cost-effectiveness did not take into account that this condition affected a minority ethnic population group with greater need for equity. Among the short term risks that NICE had to consider were that the same gene therapy, formerly called Lentiglobin, was also being tested in patients with sickle cell but due to two serious adverse reactions in patients, they had to pause their clinical trials (Terry, 2021).

However, the US Food and Drug Administration (FDA) deemed those adverse serious reactions were unrelated to the gene therapies and clinical trials with sickle cell patients resumed (Philippidis, 2021) and the gene therapy was put on the UK's Medicines and Healthcare Products Regulatory Agency's new 2021 Innovative Licensing and Access Pathway to ensure quicker access for patients to medicines but seemingly only impacting sickle cell patients (a relatively larger ethnic minority group) and not those with beta-thalassaemia where NICE is still assessing the treatment. By contrast, the European Union's Medicines Agency's Pharmacovigilance Risk Assessment Committee (PRAC) approved Zynteglo, deciding that there was a favorable benefits and risks profile and discounting some of the earlier risks presented in early trial stages (Parsons, 2021). This is still not without risks for certain patients (if they meet the conditions set out for use), as well as if conditionalities of insurance companies financing are met in the successful eradication of main symptoms of being transfusion dependent in those beta-thalassaemia patients (Urnov, 2021). However, there is also a wider perspective in understanding the interest in regulatory approvals, as such “rare” conditions represent the experimental innovative firsts in curative potentialities to be scaled up to other conditions. That is why despite a pandemic, there are political, social and financial interests in such innovation, how patients will access them and what implications they will have.

The above illustrates how local and global curative hopes are tied together but access to cures are curated by national and international bodies, patient advocacy groups as well as private and public partnerships, weighing up risks, ethics and understandings of quality of life, as well as future financial rewards to health systems by genomic innovations. However, cures are “functional” meaning that symptoms are controlled or eradicated but people still carry the gene which, for instance, has reproductive, social, cultural and psychological implications. Likewise, while treatments like gene editing (See Frangoul et al., 2021) represent a curative revolution, we do not know much how patients involved in those trials and treatments experience cure and describe it nor when such innovations will benefit those in the Global South. In that sense, we are also at the start of a revolution of genomic cures and their unequal impacts on differing groups of patients and communities.

## Discussion

We do not directly investigate cure itself nor describe it in general terms in sociology and anthropology, as research on diagnosis, personalized medicine and what happens to identity, through patient advocacy, in a lab or in the clinic while cure takes place or in prognosis, are prioritized. I have taken a step back from the precision evident in the vast literature by noting how an omission develops around “cure” and what it means empirically to undergo differing forms of cures. In the above, I have taken cure seriously as a concept to be investigated and have outlined how a logic of cure operates through three short case studies. It was noted how the act of curation or sorting through who or what will be cured or how certain diseases, disabilities or illnesses become curable or incurable biomedically, for national state interests or due to regulatory bodies responsible for assessing risks and financial rewards of novel cures, ignores many of developments found in the sociological and anthropological literature. Instead, the logic of cure is an imperative and commodified according to a biopolitics of differing forms of cure; from the seemingly incurable, to partial cures and the functionally curable. A new language around cures begins to develop and reveals what cures mean and could represent socially, affectively and temporally to patients and broader society. However, it is clear too that the language and theories of ethics and rights are critically missing in how we describe cures. We noted this was being contested.

Within disability studies and disability community responses to impact of the COVID-19 pandemic, there was the development of a “bioethics of cure” (Berghs, 2021) in criticism of: (1) lack of care as cure; (2) the ableism evident in who got access to care and why; and (3) policy creation of “vulnerability” to impairment and disablement of COVID-19. Within vaccine hesitancy we also noted ambivalence between appeals to neoliberal citizenship and responsibilities of care within a racialised pandemic that was correlated to inequalities of society. Thus, a bioethics of cure is also informed experientially by a lack of local and global ethics of care and indeterminacies of cure. Kavanagh and Broom (1998) emphasized that if you wanted to understand intersection between environmental and embodied risks, it was important to work together with people at “risk” to formulate new languages as well as approaches to those environmental and socially embodied understandings. Yet, a bioethics of cure is an empirical-ethical theory that is developing from the experiential knowledge of people who do not have access to differing forms of cures (Caron-Flinterman et al., 2005), and are undertaking differing forms of “curative labour” (Cooper and Waldby, 2014) or curative risk work to stay well and protect themselves. It is also still developing socially and will have to address the rights to impairment, not to know biosocial potentiality of cure, as well as demonstrate more ethical-rights based responses to, for instance, some people's (initial) vaccine hesitancy as choice or even total curative refusals (Benston, 2016).

Epistemological and ontological understandings of embodiment during COVID-19 were also being influenced by a biological neo-reductionism. That neo-reductionism of people to their biological immune responses, influenced medical and policy understandings of who has potentiality for COVID-19, as well as could be cured, showed an urgent need for sociological and anthropological engagement with cure. The latest developments in biosociality note that we will all have several illnesses, potentialities for disabilities and risks for conditions across the life course and these are also affected by epigenetics, inequalities and intersectional identities. However, instead of such nuanced understandings of embodiment affecting the way in which vaccines were presented, they were understood as absolute cures rather than partial with need for top-ups to ensure robust immune responses. The public had sophisticated understandings of vaccines and viruses that were not being matched by explanations of cures. Likewise, there was an ambivalence around engaging in cures that were unequal and not being given to everyone, with dangerous possible consequences.

Nowhere was this ethical ambivalence illustrated more than in the latest genomic advances, like gene therapy, that are now offering real possible cures for rare genetic conditions but trialed on populations in the Global North with strong curative advocacy groups (Keller and Packel, 2013). Those cures and access to them, are carefully nationally curated and regulated, and will not immediately benefit the Global South where most people with the conditions of sickle cell and thalassaemia are located. The emphasis there has also been on private and public partnerships with financial rewards of curing a rare disease rather than on what that entails socially, culturally, ethically, financially and biologically for patients and their families. While cure is now being described and viewed as “functional”, it is still seen in isolation from a life-course perspective where there will be numerous illnesses, diseases and conditions needing care, and where you live in the world will affect how you understand the impact of what a cure or even therapy could have. Hacking (2006) stated that we would have people coming together in terms of new types of genetic risks, and I argue that we will have new forms of identity emerging in terms of curative potentials and how those unsettle epistemologies and ontologies of the body, identity, embodiment and environment.

## Conclusion

In this article, I have set out the beginnings of a sociological and anthropological exploration of curative imperative as new normal, as well as bioethics of cure which has not been adequately examined in the literature despite understandings of how cure is changing. I have elucidated some key conceptual ideas, for example, that there is a logic of cure, connected to why epistemological and ontological positions are now at stake which debunk ideas about “biological determinism” and introduce complexities into former absolute ideas of cure toward understandings of curing. I argued that we have to examine what undergoing cure means and how hopes are invested in new technologies of cure but also understand concerns of patients or entire populations before, during and after they undergo different types of “cure” or are being “curated” as lacking certain curative potentialities. Throughout the paper, I have pointed to how cure links into categories of intersectionality that are not fixed biologically, and why they are stake in terms of how care and cure are connected to each other in the Global North and South.

## Author Contributions

The author confirms being the sole contributor of this work and has approved it for publication.

## Conflict of Interest

The author declares that the research was conducted in the absence of any commercial or financial relationships that could be construed as a potential conflict of interest.

## Publisher's Note

All claims expressed in this article are solely those of the authors and do not necessarily represent those of their affiliated organizations, or those of the publisher, the editors and the reviewers. Any product that may be evaluated in this article, or claim that may be made by its manufacturer, is not guaranteed or endorsed by the publisher.
